# Simultaneous/Selective Detection of Dopamine and Ascorbic Acid at Synthetic Zeolite-Modified/Graphite-Epoxy Composite Macro/Quasi-Microelectrodes

**DOI:** 10.3390/s130607296

**Published:** 2013-06-03

**Authors:** Elida Cristina Ilinoiu, Florica Manea, Pier Andrea Serra, Rodica Pode

**Affiliations:** 1 Department of Applied Chemistry and Engineering of Inorganic Compounds and Environment, Politehnica University of Timisoara, Victoriei Sqr., No. 2, Timisoara 300006, Romania; E-Mails: elida.ilinoiu@chim.upt.ro (E.C.I.); rodica.pode@chim.upt.ro (R.P.); 2 Department of Clinical and Experimental Medicine, University of Sassari, Viale. San Pietro 43/b, Sassari 07100, Italy; E-Mail: paserra@uniss.it

**Keywords:** dopamine, ascorbic acid, voltammetric detection, amperometric detection, synthetic zeolite-modified graphite-epoxy composite, macroelectrode, quasi-microelectrode

## Abstract

The present paper aims to miniaturize a graphite-epoxy and synthetic zeolite-modified graphite-epoxy composite macroelectrode as a quasi-microelectrode aiming *in vitro* and also, envisaging *in vivo* simultaneous electrochemical detection of dopamine (DA) and ascorbic acid (AA) neurotransmitters, or DA detection in the presence of AA. The electrochemical behavior and the response of the designed materials to the presence of dopamine and ascorbic acid without any protective membranes were studied by cyclic voltammetry and constant-potential amperometry techniques. The catalytic effect towards dopamine detection was proved for the synthetic zeolite-modified graphite-epoxy composite quasi-microelectrode, allowing increasing the sensitivity and selectivity for this analyte detection, besides a possible electrostatic attraction between dopamine cation and the negative surface of the synthetic zeolite and electrostatic repulsion with ascorbic acid anion. Also, the synthetic zeolite-modified graphite-epoxy composite quasi-microelectrode gave the best electroanalytical parameters for dopamine detection using constant-potential amperometry, the most useful technique for practical applications.

## 1. Introduction

Carbon materials in the forms of graphite, glassy carbon, carbon fibers, carbon nanotubes, *etc.*, have been important players in solid electrode development [1–3], but considering carbon composites (CC), in general, their properties present improvements over conventional solid carbon electrodes due to some interesting advantages: (i) their mouldability, which allows the construction of sensors in different configurations and sizes; (ii) the polymer matrix providing mechanical and chemical stability of the composite; (iii) low background current; (iv) microelectrode behavior given by the conductive load [4] and (v) easily miniaturized with good compatibility for *in vivo* electroanalysis of neurotransmitters [5,6].

The main drawback with carbon composites is their slow electron transfer kinetics due to the presence of the non-conducting binder, which can be solved by the incorporation of specific catalytic materials in the composite matrix. The mixture of zeolite and graphite has been proposed in various forms since the 1980s [7], leading to a class of electrodes named zeolite-modified electrodes (ZMEs) [8]. Among the class of synthetic zeolites, zeolite A is considered one of the most important molecular sieve because of its high adsorption capacity [9].

Combining sensitive materials with smaller geometries of the working electrodes, important sensing devices can be obtained for the monitoring of compounds in the cells of living organisms, of which the simultaneous determination of ascorbic acid and dopamine can serve as a typical example.

In this context, Wang and Walcarius [10] achieved a selective detection of dopamine in the presence of ascorbic acid by using a carbon paste electrode modified by zeolite by Y type, where the zeolite acted as a permselective substrate for the positively charged species, without need of any additional membrane coated on the electrode surface. This work serves as reference point for reducing geometrical area of sensing devices for determination of dopamine and other neurotransmitters.

Although, miniaturization leads to some important advantages, e.g., decreased ohmic drop of potential [11], establishment of a steady state signal [12], useful current increase [13], increased signal-to noise ratio, a conflict arises when sensitivity and selectivity are to be considered jointly, due to the fact that when improving sensitivity, the selectivity is usually decreased.

The present paper focuses on the miniaturization of a graphite-epoxy and synthetic zeolite-modified graphite-epoxy composite macroelectrode as a quasi-microelectrode aiming at *in vitro* and also, envisaging *in vivo* simultaneous electrochemical detection of dopamine and ascorbic acid neurotransmitters, or dopamine detection in the presence of ascorbic acid. The electrochemical behavior and the response of the designed materials in direct relation with their size to the presence of ascorbic acid and dopamine without any protective membranes were studied by votammetric and amperometric techniques. To the best of our knowledge, no synthetic zeolite-modified quasi-microsensors suitable for DA detection in the presence of AA have been reported before.

## 2. Experimental Section

### 2.1. Chemicals and Reagents

Synthetic graphite powder used in this study was provided from Sigma-Aldrich (Milan, Italy), with particle dimension <20 μm. A three-component epoxy resin, epoxy resin (Araldite M), epoxy hardener (Araldite M Hardener 964) and epoxy accelerator (Araldite M Accelerator 960) were purchased from Sigma-Aldrich and used as received. Natural zeolite was used as Si source for synthetic zeolite preparation using sodium hydroxide solution and sodium aluminate as aluminium source in accordance with a previously reported method [14]. The surface morphology of synthetic zeolite revealed a more uniform structure with smooth edged lamellar crystals. The particles size of the synthetic zeolite A was in the range between 20 and 300 nm and the Si/Al ratio was 1.0–1.14.

L-Ascorbic acid (Sigma, Aldrich) solutions were freshly prepared prior to each experiment and dissolved in 0.01 M HCl solution, as well as protected against light exposure during experiments.

Phosphate saline buffer solution (PBS) supporting electrolyte was prepared from dihydrogenphospate, sodium chloride and sodium hydroxide of analytical reagent grade (Sigma-Aldrich). Dopamine-hydrochloride (Sigma-Aldrich) solutions were freshly prepared prior to each experiment, dissolved in PBS and protected from light exposure during the experimental procedures. Potassium ferrocyanide and potassium ferricyanide were purchased from Sigma-Aldrich.

### 2.2. Apparatus and Experimental Measurements

#### For Macroelectrodes

Experimental investigations were carried out using an AUTOLAB potentiostat/galvanostat PGSTAT302 (EcoChemie, Utrecht, The Netherlands), controlled by a PC and the General Purpose Electrochemical System (GPES) software ver. 4.9. All the electrochemical measurements were carried out in a Metrohm three electrode cell equipped with working electrode, a platinum counter electrode and respectively, a Ag/AgCl reference electrode. Before using, working electrode was first cleaned mechanically using fine abrasive paper, 0.3 mm alumina powder (Metrohm, Herisau, Switzerland), then was washed with distilled water for 5 min.

#### For Quasi-Microelectrodes

All voltammetric experiments were carried out at room temperature (25 °C) using an eDAQ QuadStat four-channel potentiostat (Denistone East, Australia) controlled with an eChem software for cyclic voltammetry in a three-electrode electrochemical cell, carbon composite working electrodes, Ag/AgCl reference electrode and Pt auxiliary electrode. A magnetic stirrer provided the convective transport during the voltammetric experiments.

For detection experiments, the 50 mM PBS supporting electrolyte at pH 7.4 was placed in the cell, and solutions of ascorbic acid and dopamine were employed by the standard addition method. The scan rate employed for the cyclic voltametric detection experiments for quasi-microelectrodes was 250 mV·s^−1^ and 50 mV·s^−1^ for macroelectrodes.

### 2.3. Sensor Design and Construction

**Figure 1.**
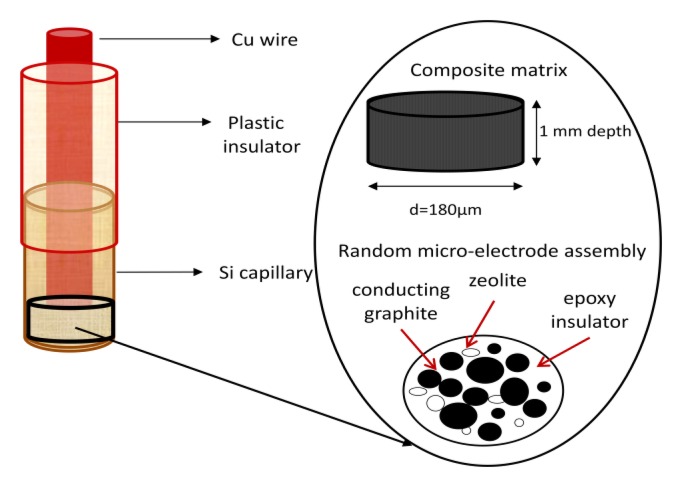
Sensor design.

The design of the quasi-microsensor is presented in Figure 1. It was made using a Classical Multi Core Cu wire (5 cm in length, i.d. = 150 μm). Taking into account the rigorous definition of the microelectrode in relation with geometrical size, the internal diameter of our sensor is slight larger than 100 μm. Due to practical reasons regarding its construction reproducibility with the capability for *in vivo* applications, this sensor is named a quasi-microsensor [15]. Its plastic insulator was removed at one edge and 2.2 cm of Cu wire was exposed and inserted in a fused silica tube (i.d. = 180 μm). At one end, 1 mm of silica tube was left free in order to allow the filling with the graphite-epoxy composite, while the Cu wire guaranteed the electrical contact with the composite matrix. The geometrical area of the sensor surface with disk geometry was 2.5 × 10^−4^ cm^2^ and the final length of the sensor used for all electrochemical detections was approximately 5 cm.

The design of the macrosensor was similar, using a Cu wire as electrical contact inserted in an PVC tube (i.d. = 2.5 cm). The geometrical area of the macrosensor surface with disk geometry was 0.196 cm^2^.

Graphite-epoxy (μ-GEC) and synthetic zeolite-modified graphite-epoxy composite (μ-SZ-GEC) quasi-microelectrodes were constructed and optimized according to Reference [16]. Graphite-epoxy (M-GEC) and synthetic zeolite-modified graphite-epoxy (M-SZ-GEC) disk macroelectrodes were used for comparison with the quasi-microelectrodes.

## 3. Results and Discussion

### 3.1. Electrochemical Surface Characterization

Cyclic voltammetry (CV) was used to determine the electroactive areas of all four M-GEC, M-SZ-GEC, μ-GEC, μ-SZ-GEC sensors by a classical potassium ferrocyanide system in 1 M KNO_3_ supporting electrolyte recorded at different scan rates using the Randles-Sevcik equation:

$${\text{I}}_{\text{p}}=2.69\times 10^{5}{\text{AD}}^{1/2}{\text{n}}^{3/2}{\text{v}}^{1/2}\text{C}$$

As can be seen in Table 1, where all coefficient values are presented, the apparent diffusion coefficients are smaller than the theoretical one (6.7 × 10^−6^ cm^2^·s^−1^) for the case of macroelectrodes (4.88 × 10^−6^ cm^2^·s^−1^ for M-GEC, 3.1 × 10^−6^ cm^2^·s^−1^ for M-SZ-GEC) and higher for the case of quasi-microelectrodes (1.13 × 10^−4^ cm^2^·s^−1^ for μ-GEC and 1.69 × 10^−5^ cm^2^·s^−1^ for -SZ-GEC, respectively).

The calculated electroactive areas for M-GEC (0.165 cm^2^) and for M-SZ-GEC (0.135 cm^2^) were lower than the geometrical area of 0.196 cm^2^, while for the quasi-microsensors the calculated electroactive areas had a higher value than the geometrical one equal with 2.5 × 10^−4^ cm^2^. The decreased electroactive surfaces for both macroelectrodes compared to their geometrical areas are dueto the distribution of graphite within the insulating materials. For the case of quasi-microsensors, the results suggest that the sensors present a large number of conducting microzones and that the majority of their surface is electroactive [17]. The responsible for this different behavior should be the various distances between the conductive islands. Probably, larger distances exist within the macroelectrode composition *versus* the quasi-microelectrodes. Also, it must be noticed that the presence of zeolite insulating material decreased further the electroactive surface area.

### 3.2. Cyclic Voltammetric Studies

It is well-known that the electrochemical detection of both dopamine (DA) and ascorbic acid (AA) in the presence of each other on untreated carbon surfaces is a problem due the the proximity of their oxidation potential values.

**Figure 2.**
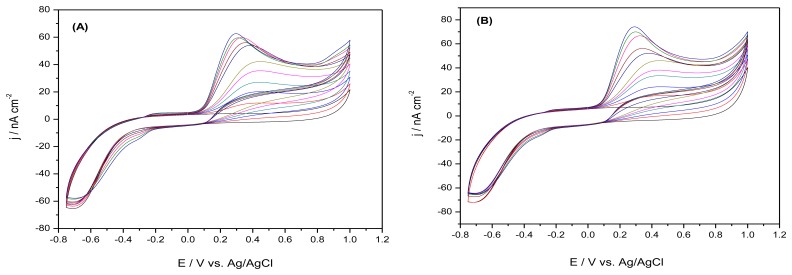
Cyclic voltammograms (CV) recorded at (**A**) M-GEC; and (**B**) M-SZ-GEC in 50 mM PBS supporting electrolyte with increasing concentrations of AA: 100 μM, 200 μM, 300 μM, 400 μM, 500 mM, followed by the addition of consecutive concentrations of DA: 10 μM, 20 μM, 30 μM, 40 μM, 50 mM; Pt reference electrode and Ag/AgCl reference electrode; potential range from −0.8 V to +1.0 V; scan rate 50 mV·s^−1^.

Figure 2 depict a series of CVs obtained for a mixture of AA and DA (consecutive concentrations of dopamine being added after consecutive additions of ascorbic acid) at the M-GEC and M-SZ-GEC macroelectrodes in the concentration range between 100 μM–500 μM for AA and between 10–50 μM for DA. The concentration range was chosen based on the *in vivo* real concentration for both targetcomponents [18]. The results show that an irreversible oxidation process appears in the first five CV curves, due to the ascorbic acid oxidation process at the carbon surface materials, while, when starting the DA standard method addition, a weak cathodic peak appears during reversed scans. These detection experiments were performed after a previous individual detection experiments at both composite materials with both molecules of interests (the results not shown here).

The electroanalytical results for both composite materials are summarized in Table 2. It can be noticed that for M-SZ-GEC composite material a better peak separation between ascorbic acid and dopamine, (*ΔE* = 0.164 V) was obtained, compared to M-GEC composite (*ΔE* = 0.147 V). Although the peak separation is less than 0.200 V, the higher value is recommended for this particular situation, and the results are promising taking into account that simultaneous detection of dopamine in the presence of ascorbic acid is very hard to achieve in normal conditions, with simple sensing devices (without improved electroactive surfaces or any protective membranes).

Based on these results, it can be observed that very close sensitivity values were obtained for the case of AA detection at both composite macrosensors (0.076 nA·μM^−1^·cm^−2^ for M-GEC and 0.075 nA·μM^−1^·cm^−2^ for M-SZ-GEC) while a significant increase of sensitivity was noticed for direct detection of DA at the M-SZ-GEC.

**Figure 3.**
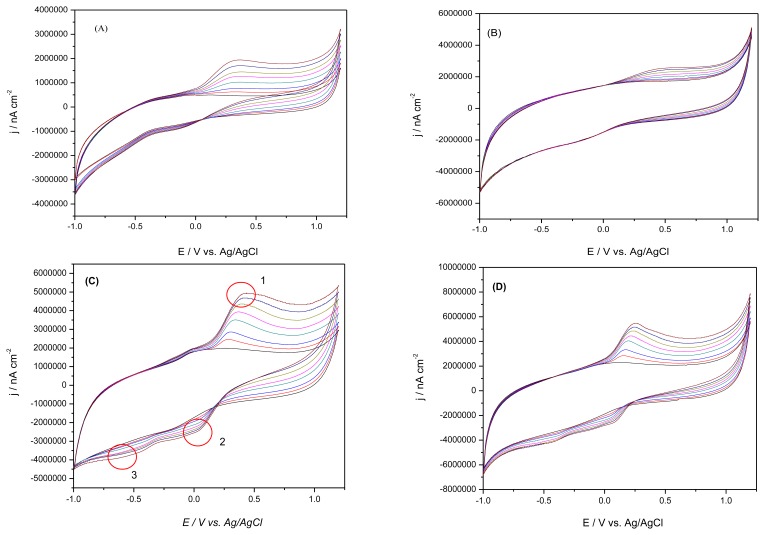
Cyclic voltammograms recorded at (**A**) μ-GEC; and (**B**) μ-SZ-GEC in 50 mM PBS supporting electrolyte with increasing concentrations of AA: 125 μM, 250 μM, 500 μM, 750 μM, 1 mM, 1.25 mM, 1.5 mM; Cyclic voltammograms recorded at; (**C**) μ-GEC and (**D**) μ-SZ-GEC in 50 mM PBS supporting electrolyte with increasing concentrations of DA: 125 μM, 250 μM, 500 μM, 750 μM, 1 mM, 1.25 mM, 1.5 mM; Pt reference electrode and Ag/AgCl auxiliary electrode; potential range from −1.0 V to +1.2 V; scan rate 250 mV·s^−1^; Pt reference electrode and Ag/AgCl auxiliary electrode; potential range from −1.0 V to +1.2 V; scan rate 250 mV·s^−1^.

Taking into account the results for simultaneous detection (of AA and DA) at macroelectrodes, the individual detection experimental response was investigated at quasi-microelectrodes having the same composition (μ-GEC and μ-SZ-GEC). Figure 3 shows the individual detection CV curves for AA and DA at graphite-epoxy composite and graphite-synthetic zeolite-epoxy composite quasi-microelectrodes.

As we expected, for both cyclic voltammograms presented in Figure 3(A,B) no cathodic peak was observed during the negative scans, showing the same irreversible AA oxidation process at the graphite composite surface. The oxidation peak current was directly proportional to AA concentrations, and linear calibration with good correlation coefficients were obtained in the range 125 μM–1.5 mM AA (Table 3). The best sensitivity of 939.47 nA·μM^−1^·cm^−2^ was obtained for the μ-GEC sensor, while a lower sensitivity in terms of possible rejection effect due to the presence of negatively charged synthetic zeolite in the composite matrix was observed for the μ-SZ-GEC sensor (554.11 nA·μM^−1^·cm^−2^). A more positive oxidation potential of AA at μ-SZ-GEC sensor (oxidation potential at 0.521 V) was observed in comparison with the bare graphite-epoxy composite quasi-microsensor (oxidation potential at 0.365 V) (see results in Table 3).

By adding increasing concentrations of DA, an increase in anodic peak oxidation is observed at the two composite microelectrodes under investigation, as can be seen in Figure 3(C,D). A considerable negative shifting of the oxidation potential is observed for the μ-SZ-GEC quasi-microelectrode, where DA occurs at 0.161 V with a clearly defined peak in comparison with the μ-GEC quasi-microelectrode (oxidation peak value at 0.281 V). Based on the DA electrochemical behaviour results produced by CV and in according with the literature data [19] the anodic peak (peak 1) corresponding to DA oxidationto *o*-quinone, and two reduction peaks corresponding to *o*-quinone reduction to DA (peak 2) and the ring closure forming leucodopaminechrome (peak 3) were identified. The last one is not good evidenced, avoiding a further leucodopaminechrome oxidation.

Under the investigated concentration range between 125 μM and 1.5 mM, the linear dependences between the anodic peak current density and DA concentration were obtained (see Table 3), response characteristic for a diffusion-controlled process, which are preferable in voltammetric determinations. The best sensitivity, 2027.35 nA·μM^−1^·cm^−2^, was also obtained for the μ-SZ-GEC quasi-microelectrode.

Under these experimental conditions, the catalytic effect towards DA oxidation was proved for μ-SZ-GEC quasi-microelectrode, where the use of zeolite modified electrodes allowed increasing the sensitivity and selectivity for this analyte detection, besides a possible electrostatic attraction between DA cation and the negative surface of the synthetic zeolite.

**Figure 4.**
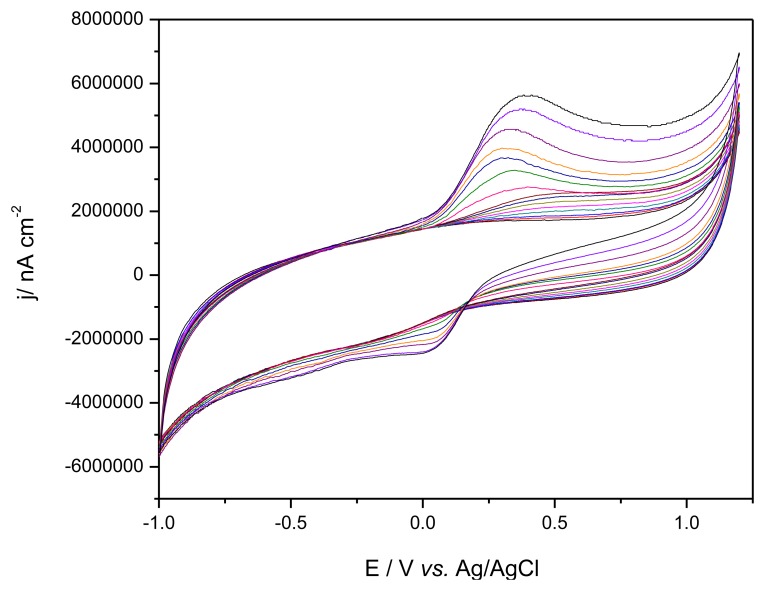
Cyclic voltammograms recorded at μ-SZ-GEC in 50 mM PBS supporting electrolyte with increasing concentrations of AA followed by increasing concentrations of DA: 125 μM, 250 μM, 500 μM, 750 μM, 1 mM, 1.25 mM, 1.5 mM; Pt reference electrode and Ag/AgCl auxiliary electrode; potential range from −0.8 V to +1.0 V; scan rate 250 mV·s^−1^.

To compare the macro and quasi-microelectrode behavior towards DA detection in the presence of AA, Figure 4 depicts a series of CVs obtained for a mixture of AA and DA (dopamine consecutive concentrations being added after consecutive additions of ascorbic acid) at μ-SZ-GEC under the similar conditions as presented in Figure 2, and the comparative results regarding the sensitivity are gathered in Table 4.

It can be noticed that the μ-SZ-GEC quasi-microelectrode allowed reaching much higher sensitivity for both AA and DA without improving peak separation. Nevertheless, by comparison of the results of DA individual and simultaneous detection (see Tables 3 and 4), the presence of AA increased only with 0.8% the voltammetric signal corresponding to DA detection, which is acceptable.

### 3.3. Comparative Chronoamperometric Detection Experiments

Constant-potential amperometry (CPA) is a very useful technique for practical applications and offers the best temporal resolution among available techniques. Taking into consideration that in the extracellular fluid of the central nervous system, DA concentrations are present at a level from nanomolar to micromolar range (0.01–1 μM) CPA should regarded as the most viable.

**Figure 5.**
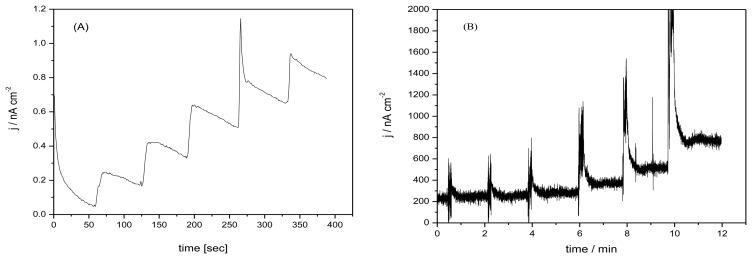
CPA recoded at (**A**) M-SZ-GEC in 50 mM PBS supporting electrolyte with adding 500 μM AA followed by increasing concentrations of Dopamine: 10 μM, 20 μM, 30 μM, 40 μM, 50 μM, Pt reference electrode and Ag/AgCl auxiliary electrode; Potential value of +0.100 V; (**B**) CPA recoded at SZ-GEC microelectrode without Nafion film in 20 mL of 50 mM PBS supporting electrolyte with increasing concentrations of Dopamine: 25 nM, 50 nM, 100 nM, 250 nM, 500 nM, 750 nM, 1 μM; Pt reference electrode and Ag/AgCl auxiliary electrode; Potential value +0.150 V.

Figure 5 presents the amperometric measurements recorded at an applied potential of +0.150 V *vs.* Ag/AgCl on M-SZ-GEC macroelectrode by continuous adding 10 μM DA (Figure 5(A)) in comparison with the amperometric signal recorded at the same potential value on μ-SZ-GEC quasi-microelectrode (Figure 5(B)) by continuous addition of 25 nM DA. No amperometric signal corresponding to the same DA concentration range as recorded with the μ-SZ-GEC was found on the M-SZ-GEC macroelectrode. Moreover, no linear dependence between amperometric response and DA concentration was achieved on the M-GEC macroelectrode, probably due to the electrode fouling occurred at DA concentrations higher than 30 μM DA. All electroanalytical parameters recorded with CPA are gathered in Table 5. The best results were obtained for the μ-SZ-GEC microsensor with a sensitivity of 540 nA·μM^−1^·cm^−2^.

*In-vitro* detection of low dopamine concentrations must be regarded as a starting point for improving the quasi-microsensors for further *in vivo* experiments.

The electroanalytical parameters determined for μ-SZ-GEC quasi-microelectrode using CV and CA are presented in Table 6. The lowest limit of detection (LOD) was evaluated based on S/N ≥ 3. The reproducibility of the μ-SZ-GEC quasi-microelectrode using the above-mentioned techniques was evaluated for five replicates measurements of dopamine detection. The values of the relative standard deviations (RSD) show the good reproducibility of the electrode.

## 4. Conclusions

Graphite-epoxy and synthetic zeolite-modified graphite-epoxy composite macroelectrodes and quasi-microelectrodes exhibited different behaviors regarding their electroactive surface areas, which were lower than the geometrical ones for the macroelectrodes. For the composite quasi-microelectrodes, the electroactive areas were higher. The distribution graphite within the insulation epoxy resin and the distances between the conductive islands should be responsible for these behaviours.

The quasi-microelectrodes consisting of the same composition as macroelectrodes allowed achieving better sensitivities for DA detection. The sensitivity of 2,044 nA ·μM^−1^ ·cm^−2^ was achieved with synthetic zeolite-modified graphite-epoxy composite quasi-microelectrodes *versus* 0.58 nA ·μM^−1^·cm^−2^, which was obtained at the same macroelectrode composition.

Under the above-presented experimental conditions, the catalytic effect towards dopamine detection was proved for the synthetic zeolite-modified graphite-epoxy composite quasi-microelectrode, allowing increased sensitivity and selectivity for detection of this analyte, besides a possible electrostatic attraction between the dopamine cation and the negative surface of the synthetic zeolite and electrostatic repulsion with ascorbic acid anion.

The results of dopamine individual and simultaneous detection on synthetic zeolite-modified graphite-epoxy composite quasi-microelectrode showed that the presence of ascorbic acid onlyincreased by 0.8% the voltammetric signal corresponding to dopamine detection, which means that ascorbic acid did not interfere the dopamine detection. Also, synthetic zeolite-modified graphite-epoxy composite quasi-microelectrode gave the best electroanalytical parameters for dopamine detection using constant-potential amperometry, the most useful technique for the practical applications.

## Figures and Tables

**Table 1.** Apparent diffusion coefficients and electroactive surface areas of GEC and SZ-GEC macroelectrodes and quasi-microelectrodes.

**Electrode Type**	**Macroelectrode**				**Quasi-Microelectrode**			
	
	**Apparent Diffusion Coefficient (cm^2^·s^−1^)**	**Theoretical Diffusion Coefficient (cm^2^·s^−1^)**	**Electro-Active Area (cm^2^)**	**Geometrical Area (cm^2^)**	**Apparent Diffusion Coefficient (cm^2^·s^−1^)**	**Theoretical Diffusion Coefficient (cm^2^·s^−1^)**	**Electro-Active Area (cm^2^)**	**Geometrical Area (cm^2^)**
GEC	4.88 × 10^−6^	6.7 × 10^−6^	0.165	0.196	1.13 × 10^−4^	6.7 × 10^−6^	1.03 × 10^−3^	2.5 × 10^−4^
SZ-GEC	3.1 × 10^−6^		0.135		1.69 × 10^−5^		3.8 × 10^−4^	

**Table 2.** Analytical parameters achieved by CV technique for DA (concentration range between 10–50 μM) and AA (concentration range between 100–500 μM) simultaneous detection experiments.

**Electrode Type**	**AA Detection**			**DA Detection**		
	
	**Sensitivity [nA·μM^−1^·cm^−2^]**	**E [V, Ag/AgCl]**	**Correlation Coefficient, R^2^**	**Sensitivity [nA·μM^−1^·cm^−2^]**	**E [V, Ag/AgCl]**	**Correlation coefficient, R^2^**
M-GEC	0.076	0.444	0.997	0.34	0.297	0.902
M-SZ-GEC	0.075	0.457	0.996	0.58	0.293	0.990

**Table 3.** Analytical parameters of the individual detection of DA (concentration range between 125 μM–1.5 mM) and AA (concentration range between 125 μM–1.5 mM) at quasi-microsensors using CV.

**Electrode Type**	**AA Detection**			**DA Detection**		
	
	**Sensitivity [nA·μM^−1^·cm^−2^]**	**E [V, Ag/AgCl]**	**Correlation Coefficient, R^2^**	**Sensitivity [nA·μM^−1^cm^−2^]**	**E [V, Ag/AgCl]**	**Correlation Coefficient, R^2^**
μ-GEC	939.47	0.365	0.999	1923.37	0.281	0.981
μ-SZ-GEC	554.11	0.521	0.998	2027.35	0.161	0.976

**Table 4.** Analytical parameters using CV techniques for simultaneous AA and DA detection at SZ-GEC macro and quasi-microelectrode.

**Analyte**	**Macroelectrode**			**Quasi-Microelectrode**		
	
	**Sensitivity [nA·μ M^−1^·cm^−2^]**	**E [V]**	**Correlation Coefficient, R^2^**	**Sensitivity [nA·μM^−1^·cm^−2^]**	**E [V]**	**Correlation Coefficient, R^2^**
AA	0.075	0.457	0.996	512	0.293	0.997
DA	0.58	0.293	0.990	2044	0.385	0.994

**Table 5.** Analytical parameters obtained with CPA techniques for DA concentration range between 10 and 50 μM at macroelectrodes and between 25 nM and 1 μM at quasi-microelectrodes.

**Electrode Type**	**Macroelectrode**			**Quasi-Microelectrode**		
	
	**Sensitivity [nA·μM^−1^·cm^−2^]**	**E [V]**	**Correlation Coefficient, R^2^**	**Sensitivity [nA·μM^−1^·cm^−2^]**	**E [V]**	**Correlation Coefficient, R^2^**
GEC	-*	0.100	-	197.2	0.150	0.989
SZ-GEC	0.078		0.999	540	0.150	0.999

* No linear dependence was found.

**Table 6.** Analytical parameters obtained for DA detection at μ-SZ-GEC quasi-microelectrode using CV and CPA.

**Technique**	**Concentration Range [μM]**	**Sensitivity [nA·μM^−1^·cm^−2^]**	**LOD [μM]**	**RSD [%]**	**Correlation Coefficient, R^2^**
CV	125–1500	2044	10	3.1	0.994
CPA	0.01–1	540	0.002	2.7	0.999
